# Association Between Social Media Use and Vaping Among Florida Adolescents, 2019

**DOI:** 10.5888/pcd18.200550

**Published:** 2021-05-13

**Authors:** Juhan Lee, Andy S.L. Tan, Lauren Porter, Kelly C. Young-Wolff, Lisa Carter-Harris, Ramzi G. Salloum

**Affiliations:** 1 Department of Health Education and Behavior, College of Health and Human Performance, University of Florida, Gainesville, Florida; 2 Annenberg School for Communication, University of Pennsylvania, Philadelphia, Pennsylvania; 3 Leonard Davis Institute of Health Economics, University of Pennsylvania, Philadelphia, Pennsylvania; 4 Bureau of Tobacco Free Florida, Florida Department of Health, Tallahassee, Florida; 5 Division of Research, Kaiser Permanente Northern California, Oakland, California; 6 Department of Psychiatry and Behavioral Sciences, Memorial Sloan Kettering Cancer Center, New York, New York; 7 Department of Health Outcomes and Biomedical Informatics, College of Medicine, University of Florida, Gainesville, Florida

## Abstract

**Introduction:**

With the growing popularity of vaping, evidence has emerged about the association between social media use and vaping among adolescents, possibly because of the proliferation of e-cigarette advertisements and other related content on social media. Our study examined the association between social media use and vaping among adolescents.

**Methods:**

Using data from the 2019 Florida Youth Tobacco Survey (N = 10,776), we conducted logistic regression models on adolescent vaping status (experimental and current vaping) by nondaily and daily use of social media platforms — Facebook, Instagram, Twitter and Snapchat, controlling for other confounders.

**Results:**

Use of all 4 selected social media platforms was significantly associated with vaping status (*P* <.001 for all). Once jointly analyzed, daily use of Instagram was significantly associated with increased relative risks of experimental (adjusted relative risk ratio [aRRR] = 1.76; 95% CI, 1.38–2.25) and current vaping (aRRR = 1.51; 95% CI, 1.16–1.95); nondaily use of Snapchat was significantly associated with increased relative risk of experimental (aRRR = 1.57; 95% CI, 1.17–2.10) and current vaping (aRRR = 1.87; 95% CI, 1.31–2.66); daily use of Snapchat was associated with increased relative risk of experimental (aRRR = 2.38; 95% CI, 1.85–3.08) and current vaping (aRRR = 5.09; 95% CI, 3.78–6.86); nondaily use of Facebook was associated with increased relative risk of current vaping (aRRR = 1.20; 95% CI, 1.00–1.43), and nondaily use of Twitter was associated with increased relative risk of current vaping (aRRR = 1.29; 95% CI, 1.07–1.56).

**Conclusion:**

Multilevel efforts are warranted to monitor social media use and vaping status among adolescents, including media use monitoring plans, developing counter-marketing campaigns, and strict regulatory action on social media.

**Summary**

**What is already known on this topic?**

With the growing popularity of vaping and the proliferation of e-cigarette advertisements on social media, evidence has emerged about the association between social media exposure and vaping.

**What is added by this report?**

This study highlights the potentially stronger influence of Snapchat (daily and nondaily), Instagram (daily use), Facebook (nondaily use), and Twitter (nondaily use), on experimental and current vaping among adolescents in Florida.

**What are the implications for public health practice?**

Public health interventions are needed to promote education and brief counseling about social media use in schools, households, and clinics. In addition, it is important to develop counter-marketing campaigns targeting social media platforms that adolescents most frequently use (eg, Instagram, Snapchat), strict age restrictions, and regulatory actions at the policy level.

## Introduction

With the growing popularity of vaping (1), evidence has emerged about the association between social media use and adolescent vaping (2). This association may be attributed to the proliferation of e-cigarette advertisements and other related content on social media (3). Advertisements on social media may pose particular risks because of high levels of social media use by adolescents (4) and unregulated marketing messages that appeal to adolescents (5). For example, e-cigarette advertisements on social media may be individualized based on a user’s demographic characteristics or search histories (6). Moreover, social media users can share e-cigarette advertisements with members of their social network, leading to rapid dissemination across populations (6).

Besides serving as a platform for e-cigarette advertisements, social media sites facilitate information sharing about e-cigarettes with images of their use among adolescents (7). As such, social media platforms provide opportunities for adolescents to acquire new information about e-cigarette use and behavior (8,9). For example, social media platforms such as YouTube provide tutorials with basic information about how to use e-cigarettes and vaping products (10). Furthermore, seeing peers or influencers (11) use e-cigarettes on social media may promote the perception among adolescents that e-cigarette use is a socially accepted behavior (12). Exposure to e-cigarette–related content on social media, therefore, might be associated with susceptibility to e-cigarette use among adolescents.

Several studies found an association between social media exposure and e-cigarette use among adolescents (6,12) and young adults (13,14). A recent study found that a higher level of social media use among adolescents was associated with their greater susceptibility to use e-cigarettes, as well as positive attitudes and perceptions of low harm related to e-cigarette use (12). Only a few studies, however, have examined which social media platforms most influence adolescent vaping behaviors. Among adolescents in Connecticut, exposure to e-cigarette advertisements on Facebook was associated with e-cigarette use initiation beyond other advertisement channels (6). A recent study surveyed college students and found a higher daily use of Snapchat (80%) than other social media platforms (Instagram, 73%; YouTube, 59%; Facebook, 54%) (15). That study also found only Snapchat use, not use of other platforms, was associated with higher odds of ever vaping among college students (16). That study, however, was limited to college students, and did not include adolescents. Given the rapidly evolving social media landscape (17), recent population-based surveillance tools should be examined to understand which platforms are most influential in adolescent e-cigarette use to inform implementation of effective counter-marketing strategies on social media.

Our study investigated the relationship between the use of 4 social media platforms (Facebook, Instagram, Twitter, and Snapchat) and vaping status among Florida adolescents. We hypothesized that 1) the use of each social media platform is associated with adolescent vaping, and 2) social media platforms that are more popular among adolescents (eg, Snapchat) (17) have stronger associations with adolescent vaping.

## Methods

We analyzed data from the 2019 Florida Youth Tobacco Survey (FYTS), an annual cross-sectional statewide, school-based survey that uses a multistage stratified sample design representative of middle and high school students in Florida (N = 10,776) (18). Weighted response rates were 71.6% for middle school students and 68.6% for high school students. We included respondents who reported e-cigarette use (vaping) status in the analytic sample (n = 10,475).

Respondents were asked about their vaping experiences (ie, ever used vaping products and products vaped in the past 30 days). We categorized products as 1) never vaped, 2) experimentally used, but not currently (ever vaped), and 3) currently vaping. Ever vaped was defined as vaping product ever used, but not in the past 30 days, and currently vaping was defined as vaping product ever vaped and used at least 1 day in the past 30 days.

We also asked respondents how often they visited each social media platform (Facebook, Instagram, Twitter, Snapchat). The response options were never, every few months, every few weeks, 1–2 days per week, 3–5 days per week, once per day, and several times per day. For respondent use of each social media platform, we categorized responses as 1) never, 2) nondaily, and 3) daily.

For potential confounders, we used grade level (middle or high school), sex (female or male), race/ethnicity (non-Hispanic White, non-Hispanic Black, Hispanic, or non-Hispanic other), current cigarette smoking (yes or no), current other tobacco use besides cigarettes and e-cigarettes (any or none), familial use of any tobacco product (any or none), and past 12-month internalizing tendencies (eg, feeling sad and hopeless, yes or no). We included internalizing symptoms because they may be associated with vaping status (19) and social media use (8,20) among adolescents.

### Statistical analyses

We estimated the prevalence of e-cigarette use by each social media platform and examined associated factors. The Rao–Scott adjusted χ^2^ test was used to examine significant associations between variables. We first conducted unadjusted multinomial logistic regression to examine the bivariate association between each social media platform used and vaping status. We jointly analyzed associations between the use of the 4 social media platforms and vaping status, controlling for respondent characteristics. Because e-cigarette and social media use patterns may be different in middle and high school students (21,22), we further estimated multivariable, multinomial logistic regression models, stratified by middle school students versus high school students. We used the *svy* commands in Stata 16.0 (StataCorp LLC) to accommodate the complex sampling design of the FYTS (18). We used *P* < .05 as the cutoff for significance. This observational study was deemed exempt by the University of Florida Institutional Review Board.

## Results

The analytic sample (n = 10,475) was composed mostly of high school students (56.9%, weighted); 50.4% were male, 38.8% were non-Hispanic White, and 33.5% were Hispanic. Of current e-cigarette users, 71.6% were current cigarette smokers, 73.2% were current other tobacco product users, 26.3% reported family members using tobacco products, and 24.8% of adolescents reported internalizing symptoms (eg, depressive symptoms) in the past 12 months (Table 1). Of the analytic sample, 13.7% (weighted; unweighted n = 1,434) reported experimental use, and 15.9% (n = 1,673) reported current vaping. The prevalence of current vaping was higher among daily Instagram users (20.6%) than among nondaily users (11.9%) and never users of Instagram (6.1%). The prevalence of current vaping was also higher among daily Snapchat users (22.5%) than among nondaily users (9.5%) and never users of Snapchat (4.1%). We observed similar patterns among Facebook and Twitter users.

**Table 1:** Prevalence in Vaping Status by Social Media Use and Associated Factors Among Adolescents, 2019 Florida Youth Tobacco Survey

Platform	Use (n)	Vaping Status, Weighted % (95% CI)^a^			*P* Value^d^
		Never Used (n = 7,368)	Experimentally Used (n = 1,434)^b^	Current Use (n = 1,673)^c^	
**Social Media Use**					
**Facebook**					
Never	6,057	74.6 (72.2–76.8)	12.7 (11.5–13.9)	12.8 (11.3–14.5)	<.001
Nondaily^e^	1,983	60.7 (57.8– 63.5)	16.6 (14.8–18.5)	22.8 (20.5–25.2)	
Daily^f^	1,118	58.8 (55.4–62.1)	18.3 (15.8–21.0)	22.9 (20.1–26.0)	
**Instagram**					
Never	2,007	87.0 (84.9–88.9)	6.8 (5.6–8.4)	6.1 (5.0–7.5)	<.001
Nondaily^e^	1,225	76.1 (73.0–78.9)	12.0 (10.0–14.4)	11.9 (10.0–14.1)	
Daily^f^	6,011	62.3 (59.8–64.8)	17.1 (15.8–18.4)	20.6 (18.7–22.7)	
**Twitter**					
Never	6,008	74.6 (72.3–76.8)	12.3 (11.1–13.6)	13.1 (11.6–14.8)	<.001
Nondaily^e^	1,667	61.2 (58.0–64.3)	17.5 (15.3–19.9)	21.3 (19.0–23.9)	
Daily^f^	1,453	57.5 (53.8–61.2)	18.8 (16.6–21.2)	23.7 (21.0–26.7)	
**Snapchat**					
Never	2,230	89.1 (87.5–90.6)	6.8 (5.7–8.1)	4.1 (3.2–5.1)	<.001
Nondaily^e^	1,279	77.3 (74.6–79.9)	13.1 (11.3–15.3)	9.5 (7.9–11.4)	
Daily^f^	5,708	60.2 (57.6–62.7)	17.3 (16.0–18.8)	22.5 (20.5–24.6)	
**Associated Factors**					
**Grades**					
Middle school (grades 6–8)	4,935	81.3 (79.6–83.0)	9.6 (8.7–10.6)	9.0 (7.9–10.4)	<.001
High school (grades 9–12)	5,521	60.5 (58.1–62.9)	17.4 (16.2–18.7)	22.1 (20.1–24.3)	
**Sex**					
Female	5,326	68.8 (66.3–71.2)	14.1 (12.9–15.4)	17.1 (15.4–19.0)	.23
Male	5,031	70.2 (67.7–72.5)	14.1 (12.9–15.3)	15.8 (14.0–17.7)	
**Race/ethnicity**					
Non-Hispanic White	3,426	64.8 (61.3–68.1)	14.5 (12.8–16.4)	20.7 (18.4–23.3)	<.001
Non-Hispanic Black	1,824	77.3 (74.6–79.8)	13.2 (11.4–15.3)	9.5 (8.0–11.1)	
Hispanic	4,164	69.2 (66.5–71.7)	14.4 (13.2–15.8)	16.4 (14.6–18.3)	
Non-Hispanic others	822	72.6 (68.7–76.1)	13.6 (11.1–16.5)	13.9 (11.2–17.0)	
**Current cigarette smoking**					
No	10,221	70.7 (68.5–72.9)	14.1 (13.1–15.1)	15.2 (13.7–16.9)	<.001
Yes	208	15.7 (11.0–22.0)	12.7 (8.2–19.3)	71.6 (63.7–78.3)	
**Current other tobacco use**					
No	9,657	73.0 (70.8–75.0)	13.9 (12.9–15.0)	13.1 (11.7–14.7)	<.001
Yes	541	12.6 (9.7–16.2)	14.2 (11.3–17.7)	73.2 (68.5–77.5)	
**Familial tobacco use^g^**					
No	6,266	76.6 (74.3–78.8)	12.2 (11.1–13.5)	11.2 (9.8–12.7)	<.001
Yes	3,225	55.7 (52.8–58.6)	18.0 (16.4–19.6)	26.3 (23.9–28.9)	
**Internalizing tendency (eg, depressive symptom)^h^**					
No	6,561	74.6 (72.2–76.9)	12.3 (11.2–13.6)	13.0 (11.4–14.8)	<.001
Yes	2,672	56.2 (53.5–58.8)	19.1 (17.4–20.8)	24.8 (22.6–27.0)	

a Accounted for complex sampling design of Taylor series linearization as variance estimation.

b Vaped, but not vaped in the past 30 days.

c Vaped in the past 30 days.

d Estimated using Rao-Scott adjusted χ^2^ test.

e Included every few months, every few weeks, 1–2 days/week, and 3–5 days/week.

f Included once per day and several times per day.

g Assessed using the question, Does anyone who lives in your home use any of the following products? (Do not count yourself), and response options were cigarettes, cigars, chewing tobacco, snuff, or dip, hookah, and electronic vaping products.

h Assessed using the question, During the past 12 months, did you ever feel so sad or hopeless that for ≥2 consecutive weeks you stopped usual activities?

In unadjusted models, nondaily and daily use of all 4 social media platforms were significantly associated with increased relative risks of experimental and current vaping versus never use (*P* < .001 for all). In the adjusted models that included use of all social media platforms as predictors and controlling for respondent characteristics, daily use of Instagram was significantly associated with increased relative risks of experimental use (adjusted relative risk ratio [aRRR] = 1.76; 95% CI, 1.38–2.25) and current vaping (aRRR = 1.51; 95% CI, 1.16–1.95); nondaily use of Snapchat was significantly associated with increased relative risks of experimental use (aRRR = 1.57; 95% CI, 1.17–2.10) and current vaping (aRRR = 1.87; 95% CI, 1.31–2.66); daily use of Snapchat was associated with increased relative risks of experimental use (aRRR = 2.38; 95% CI, 1.85–3.08) and current vaping (aRRR = 5.09; 95% CI, 3.78–6.86); nondaily use of Facebook was associated with increased relative risks of current vaping (aRRR = 1.20; 95% CI, 1.00–1.43), and nondaily use of Twitter was associated with increased relative risk of current vaping (aRRR = 1.29; 95% CI, 1.07–1.56) (Table 2).

**Table 2:** Results of Adjusted and Unadjusted Multinomial Logistic Regression Models for Four Social Media Platforms Among Adolescents in Florida, 2019^a^

Platform Use	Unadjusted RRR (95% CI)^b^		Adjusted RRR (95% CI)^c^	
	Experimental Vaping^d^ (n = 1,434)	Current Vaping^e^ (n = 1,673)	Experimental Vaping^d^ (n = 1,434)	Current Vaping^e^ (n = 1,673)
**Facebook**				
Never^f^	1 [Reference]	1 [Reference]	1 [Reference]	1 [Reference]
Nondaily^g^	1.61 (1.37–1.90)	2.19 (1.88–2.55)	1.03 (0.86–1.23)	1.20 (1.00–1.43)
Daily^h^	1.83 (1.51–2.22)	2.27 (1.90–2.71)	1.04 (0.84–1.29)	0.93 (0.73–1.18)
**Instagram**				
Never	1 [Reference]	1 [Reference]	1 [Reference]	1 [Reference]
Nondaily	2.01 (1.49–2.72)	2.21 (1.69–2.90)	1.24 (0.89–1.72)	0.93 (0.66–1.30)
Daily	3.48 (2.79–4.34)	4.70 (3.76–5.87)	1.76 (1.38–2.25)	1.51 (1.16–1.95)
**Twitter**				
Never	1 [Reference]	1 [Reference]	1 [Reference]	1 [Reference]
Nondaily	1.74 (1.46–2.08)	1.98 (1.70–2.31)	1.20 (0.97–1.48)	1.29 (1.07–1.56)
Daily	1.98 (1.62–2.43)	2.35 (2.00–2.75)	1.22 (0.98–1.51)	1.22 (0.99–1.50)
**Snapchat**				
Never	1 [Reference]	1 [Reference]	1 [Reference]	1 [Reference]
Nondaily	2.23 (1.73–2.86)	2.68 (2.00–3.60)	1.57 (1.17–2.10)	1.87 (1.31–2.66)
Daily	3.78 (3.05–4.68)	8.13 (6.36–10.38)	2.38 (1.85–3.08)	5.09 (3.78–6.86)
**Adolescent Characteristics**				
**Grades**				
Middle school (grades 6–8)	1 [Reference]	1 [Reference]	1 [Reference]	1 [Reference]
High school (grades 9–12)	2.43 (2.10–2.81)	3.28 (2.68–4.02)	2.00 (1.69–2.37)	2.45 (2.02–2.96)
**Sex**				
Female	1 [Reference]	1 [Reference]	1 [Reference]	1 [Reference]
Male	0.98 (0.87–1.10)	0.90 (0.80–1.02)	1.31 (1.14–1.50)	1.11 (0.94–1.31)
**Race/ethnicity**				
Non-Hispanic White	1 [Reference]	1 [Reference]	1 [Reference]	1 [Reference]
Non-Hispanic Black	0.77 (0.61–0.97)	0.38 (0.30–0.48)	0.75 (0.59–0.95)	0.40 (0.30–0.54)
Hispanic	0.93 (0.78–1.12)	0.74 (0.62–0.89)	0.89 (0.74–1.08)	0.75 (0.62–0.91)
Non-Hispanic others	0.84 (0.65–1.08)	0.60 (0.47–0.76)	0.76 (0.56–1.02)	0.68 (0.50–0.91)
**Current cigarette smoking**				
No	1 [Reference]	1 [Reference]	1 [Reference]	1 [Reference]
Yes	4.09 (2.26–7.40)	21.20 (13.94–32.25)	2.83 (1.13–7.11)	8.96 (3.87–20.75)
**Current other tobacco use (eg, cigars, hookah, smokeless tobacco)**				
No	1 [Reference]	1 [Reference]	1 [Reference]	1 [Reference]
Yes	5.92 (4.16–8.43)	32.38 (23.73–44.19)	4.49 (2.89–6.97)	24.40 (16.04–37.11)
**Familial tobacco use^i^**				
No	1 [Reference]	1 [Reference]	1 [Reference]	1 [Reference]
Yes	2.02 (1.75–2.32)	3.24 (2.86–3.68)	1.84 (1.58–2.14)	2.47 (2.11–2.88)
**Internalizing tendency (eg, depressive symptom)^j^**				
No	1 [Reference]	1 [Reference]	1 [Reference]	1 [Reference]
Yes	2.05 (1.79–2.35)	2.52 (2.23–2.86)	1.78 (1.53–2.06)	1.91 (1.62–2.25)

Abbreviation: RRR, relative risk ratio.

a Data source: Florida Youth Tobacco Survey.

b Unadjusted multinomial logistic regression models examined the bivariate relationship between predictors and outcome.

c Adjusted multinomial logistic regression model included all variables in 1 model.

d Vaped, but not vaped in the past 30 days.

e Vaped in the past 30 days.

f Never used e-cigarette reference group is n = 7,368.

g Nondaily includes every few months, every few weeks, 1–2 days/week, and 3–5 days/week.

h Daily includes once per day or several times per day.

i Assessed using the question, Does anyone who lives in your home use any of the following products? (Do not count yourself), and response options were cigarettes, cigars, chewing tobacco, snuff, or dip, hookahs, and electronic vaping products.

j Assessed using the question, During the past 12 months, did you ever feel so sad or hopeless that for ≥2 consecutive weeks you stopped usual activities?

Among middle school students, nondaily use of Facebook was associated with increased relative risks of current vaping (aRRR = 1.59; 95% CI, 1.13–2.23). Nondaily use (aRRR = 1.74; 95% CI, 1.07–2.85) and daily use (aRRR = 2.19; 95% CI, 1.52–3.15) of Instagram was associated with increased relative risk of experimental vaping. Nondaily use of Twitter was associated with increased relative risk of experimental vaping (aRRR = 1.45; 95% CI, 1.05–1.99). Daily use of Snapchat was associated with increased relative risk of experimental (aRRR = 1.96; 95% CI, 1.36–2.84) and current vaping (aRRR 3.80; 95% CI, 2.36–6.11) (Table 3).

**Table 3:** Results of Multivariable, Multinomial, Logistic Regression Models for Vaping Among Adolescents, 2019 Florida Youth Tobacco Survey

Social Media Use	Middle school (n = 4,935)^a^		High school (n = 5,521)^b^	
	Experimental Use (n = 486)^c^	Current Use (n = 456)^d^	Experimental Use (n = 947)	Current Use (n = 1,216)
**Adjusted relative risk ratio (95% CI)**				
**Facebook**				
Never	1 [Reference]	1 [Reference]	1 [Reference]	1 [Reference]
Nondaily^e^	1.25 (0.92–1.69)	1.59 (1.13–2.23)	0.97 (0.78–1.20)	1.10 (0.89–1.35)
Daily^f^	1.27 (0.84–1.90)	1.45 (0.87–2.39)	0.96 (0.75–1.24)	0.83 (0.63–1.08)
**Instagram**				
Never	1 [Reference]	1 [Reference]	1 [Reference]	1 [Reference]
Nondaily	1.74 (1.07–2.85)	0.89 (0.55–1.43)	0.90 (0.59–1.39)	0.89 (0.56–1.42)
Daily	2.19 (1.52–3.15)	1.31 (0.85–2.01)	1.45 (1.05–2.00)	1.56 (1.12–2.19)
**Twitter**				
Never	1 [Reference]	1 [Reference]	1 [Reference]	1 [Reference]
Nondaily	1.45 (1.05–1.99)	1.37 (0.83–2.34)	1.10 (0.85–1.42)	1.25 (1.00–1.57)
Daily	1.16 (0.77–1.76)	3.80 (2.36–6.11)	1.21 (0.94–1.55)	1.30 (1.03–1.66)
**Snapchat**				
Never	1 [Reference]	1 [Reference]	1 [Reference]	1 [Reference]
Nondaily	1.26 (0.80–1.99)	1.39 (0.83–2.34)	1.87 (1.31–2.68)	2.24 (1.41–3.56)
Daily	1.96 (1.36–2.84)	3.80 (2.36–6.11)	2.80 (2.00–3.93)	6.12 (4.24–8.83)
**Sex**				
Female	1 [Reference]	1 [Reference]	1 [Reference]	1 [Reference]
Male	1.72 (1.37–2.17)	1.09 (0.83–1.43)	1.14 (0.97–1.33)	1.08 (0.88–1.32)
**Race/ethnicity**				
Non-Hispanic White	1 [Reference]	1 [Reference]	1 [Reference]	1 [Reference]
Non-Hispanic Black	1.16 (0.80–1.68)	0.89 (0.58–1.38)	0.60 (0.46–0.80)	0.30 (0.21–0.41)
Hispanic	1.09 (0.83–1.44)	1.00 (0.73–1.36)	0.80 (0.64–1.02)	0.67 (0.53–0.85)
Non-Hispanic others	1.05 (0.67–1.63)	0.68 (0.39–1.18)	0.65 (0.45–0.96)	0.67 (0.47–0.95)
**Current cigarette smoking**				
No	1 [Reference]	1 [Reference]	1 [Reference]	1 [Reference]
Yes	2.46 (0.65–9.34)	7.45 (2.08–26.68)	3.17 (0.90–11.08)	10.16 (3.12–33.10)
**Current other tobacco use (eg, cigars, hookah, smokeless tobacco)**				
No	1 [Reference]	1 [Reference]	1 [Reference]	1 [Reference]
Yes	3.74 (1.45–9.62)	26.67 (12.80–55.56)	4.72 (2.87–7.77)	24.02 (14.57–39.58)
**Familial tobacco use^g^**				
No	1 [Reference]	1 [Reference]	1 [Reference]	1 [Reference]
Yes	2.14 (1.69–2.70)	3.03 (2.32–3.96)	1.70 (1.40–2.07)	2.27 (1.88–2.75)
**Internalizing tendency (eg, depressive symptom)^h^**				
No	1 [Reference]	1 [Reference]	1 [Reference]	1 [Reference]
Yes	2.20 (1.70–2.85)	2.54 (1.98–3.27)	1.56 (1.32–1.86)	1.69 (1.39–2.07)

a Middle school reference group is n = 4,935.

b High school referent group is n = 5,521.

c *P* < .05; 2-sided.

d Current use is defined as vaped in the past 30 days.

e Nondaily includes every few months, every few weeks, 1–2 days/week, and 3–5 days/week.

f Daily includes once per day and several times per day.

g Assessed using the question, Does anyone who lives in your home use any of the following products? (Do not count yourself), and response options were cigarettes–cigars–chewing tobacco, snuff or dip, hookahs, and electronic vaping products.

h Assessed using the question, During the past 12 months, did you ever feel so sad or hopeless that for ≥2 consecutive weeks you stopped usual activities?

Among high school students, nondaily and daily use of Facebook were not associated with experimental or current vaping status. Daily use of Instagram was associated with increased relative risk of experimental (aRRR = 1.45; 95% CI, 1.05–2.00) and current vaping (aRRR = 1.56; 95% CI, 1.12–2.19). Nondaily use (aRRR = 1.25; 95% CI, 1.00–1.57) and daily use of Twitter (aRRR = 1.30; 95% CI, 1.03–1.66) was associated with increased relative risk of current vaping. Nondaily use of Snapchat was associated with increased relative risk of experimental (aRRR = 1.87; 95% CI, 1.31–2.68) and current vaping (aRRR = 2.24; 95% CI, 1.41–3.56). Daily use of Snapchat was also associated with increased relative risk of experimental (aRRR = 2.80; 95% CI, 2.00–3.93) and current vaping (aRRR = 6.12; 95% CI, 4.24–8.83).

## Discussion

This study highlights the potential influence of Snapchat (daily and nondaily use), Instagram (daily use), Facebook (nondaily use), and Twitter use (nondaily use) on experimental and current vaping among adolescents in Florida. This association may be explained by the recent increase in popularity of social media platforms among adolescents (17) and the potential exposure to e-cigarette advertisement or e-cigarette–related content on these platforms (2).

We found that Snapchat use was more consistently associated with experimenting and current vaping, and these results were observed even after stratifying models by middle and high school. This result supports past research finding that only Snapchat use was associated with ever vaping among college students, while other platforms (eg, Facebook, Instagram, and YouTube) were not associated with vaping status (15). This finding may be explained by the unique features of Snapchat as a platform. For example, Snapchat is used for peer-to-peer messaging and sharing of pictures for a short period, and the messages and pictures subsequently disappear. Snapchat includes “stories” and “discover” features, a collection of “snaps” lasting 24 hours between users and users’ network. Content can be private and not publicly viewed, unlike other platforms where the content is mostly public and relatively easy to monitor.

Although social media sites might have policies that prohibit e-cigarette advertisements, these restrictions are not strictly enforced and might not apply to all content (24,25). The prevalence of e-cigarette advertising on social media has resulted in the US Food and Drug Administration (FDA) issuing warning letters to e-cigarette companies about influencer posts (26,27). The use of e-cigarettes has been depicted as glamorous, popular, and socially acceptable, such as through the public actions of vaping (28). Portraying peers, acquaintances, or influencers in a way that portrays them as glamorous, popular, socially acceptable, or appealing while using e-cigarettes might influence adolescents to try e-cigarettes (12). Furthermore, other portrayals of e-cigarette use on social media (ie, safe, convenient, having positive health effects from current user testimony) might also appeal to adolescents (28–30). Several social media features make it difficult to monitor marketing practices that target adolescents (24). These features include loose age restrictions on social media (25) and Snapchat’s feature video content that disappears after viewing. Developing strategies to monitor e-cigarette companies’ marketing activities and e-cigarette–related content on social media, therefore, is as important as developing counter campaigns on social media to prevent vaping among adolescents. Further, a need exists for stronger penalties and prompt enforcement on social media platforms that violate tobacco-marketing policies (31).

This study has several limitations. First, because it is based on data from a single state, generalization to US adolescents outside Florida might not be possible. Second, because we relied on self-reported measures, results might have been influenced by self-report biases, such as recall or social desirability. Third, given its cross-sectional observation design, this study cannot provide results on causality or temporality. Fourth, the FYTS did not assess other popular social media platforms, such as YouTube and TikTok. Given the recent frequent portrayal of e-cigarettes on TikTok (32), the FYTS should consider including TikTok and other popular platforms in future surveys. Fifth, it is possible that the FYTS did not explicitly evaluate other unmeasured confounders associated with both social media and vaping, such as exposure to e-cigarette advertisements, vaping content on social media, or the cost of e-cigarettes. Sixth, we did not test potential interaction effects of social media use with other associated factors such as income level (33), sexual orientation, and gender identity (34,35). Lesbian, gay, bisexual, and transgender adolescents are more likely to use social media to form communities and create a shared sense of identity (34), which in turn makes them more likely to be exposed to e-cigarette–related content on social media (35). Lastly, future inquiries should examine exposure inequities in e-cigarette advertisements on social media and the implications of this exposure.

Despite these limitations, public health interventions are needed to provide education and guidance on social media use in schools, households, and clinics (10). For example, the American Academy of Pediatrics has suggested that parents and pediatricians collaboratively develop a Family Media Use Plan (36). This plan would include household rules for media consumption (such as monitoring) tailored for different developmental stages (8). In addition, it might be important to develop counter-marketing campaigns for adolescents that target their frequently used social media platforms, such as Instagram and Snapchat, and to develop strict age restrictions on social media content and regulatory policy actions at state and federal levels (1). The Real Cost is an example of a counter-marketing campaign that was launched by the FDA in 2014 and expanded to include e-cigarette use in 2018. The goal of the campaign is to educate adolescents at high risk of smoking and vaping about the dangerous chemicals in tobacco and the harmful effects of tobacco use, such as the loss of control caused by addiction (10). The e-cigarette prevention campaign obtained 2 billion teen views at initial evaluation, reporting 578,000 likes, 89,000 shares, and 31,000 comments (37). However, vaping-related hashtags were used up to 10,000 times more often than the FDA-sponsored hashtag #TheRealCost on Instagram (37). A stronger presence, therefore, of public health counter-marketing on social media may be warranted (37). Overall, this study highlights the influence of social media use on vaping status among adolescents, specifically in 4 popular social media platforms. Multilevel efforts to monitor social media use and vaping status among adolescents are needed.
